# Supplementary material for: Genome-Wide Analysis of Factors Affecting Transcription Elongation and DNA Repair: A New Role for PAF and Ccr4-Not in Transcription-Coupled Repair
Source: PLoS Genet. 2009 Feb 6;5(2):e1000364. doi: 10.1371/journal.pgen.1000364 (PMC2629578; doi:10.1371/journal.pgen.1000364)
Supplement: Table S1 — Analysis of genes whose expression is affected by 4-NQO and MPA. Expression of a total of 2374 genes was determined by microarray analysis after treating wild-type cells with either 75 ng/ml of 4-NQO or 50 µg/ml of MPA for 30 min each. The genes showing mRNA levels that were at least 2-fold above or below mock treated cells for each treatment are listed. The complete microarray data are available at http://www.ncbi.nlm.nih.gov/geo/ under the access number GSE11561. (0.06 MB PDF) [file pgen.1000364.s002.pdf]

Supplementary Table S1

MPA:

ORF	Name	fold expression change
YML128C	MSC1	11,9
YFL014W	HSP12	6,8
YKL035W	UGP1	6,9
YNL055C	POR1	7
YGR248W	SOL4	7
YLR134W	PDC5	5,8
YKR042W	UTH1	6,8
YHR019C	DED81	7,5
YAR073W	IMD1	12,6
YKL081W	TEF4	5,9
YCL040W	GLK1	5
YLL026W	HSP104	5,8
YGL008C	PMA1	5,4
YMR096W	SNZ1	7,3
YGL215W	CLG1	7,9
YPR028W	YOP1	5,8
YLL028W	TPO1	3,8
YMR303C	ADH2	3,7
YLR432W	IMD3	8,3
YPR149W	NCE102	4,5
YHR216W	IMD2	8,3
YGL245W		3,9
YNL003C	PET8	3,6
YMR046C		4,4
YBR214W	SDS24	4,6
YJR121W	ATP2	4
YHL015W	RPS20	9,7
YAR010C		4,9
YOL041C	NOP12	5
YLR356W		3,1
YKL152C	GPM1	3,1
YAL005C	SSA1	5,5
YER081W	SER3	4,7
YGL037C	PNC1	6,1
YBL045C	COR1	3,3
YNL160W	YGP1	3,9
YBR286W	APE3	4,7
YML040W		3
YML045W		3,3
YHR008C	SOD2	4
YIR034C	LYS1	4
YDR033W	MRH1	5,2
YMR105C	PGM2	3,8
YBR263W	SHM1	3,8
YBR011C	IPP1	3,9
YHR011W	DIA4	3
YOR120W	GCY1	3,5
YMR203W	TOM40	3,3
YMR251W-A	HOR7	2,9
YMR051C		3,7
YJR026W		3,8
YBR234C	ARC40	3,5

4-NQO:

ORF	Name	fold expression change
YJR073C	OPI3	11,7
YLR178C	TFS1	6,4
YLR109W	AHP1	5,3
YHR087W		4,5
YFL014W	HSP12	4,2
YCL040W	GLK1	4,1
YMR250W	GAD1	4
YML028W	TSA1	3,9
YML131W		3,9
YBL064C	PRX1	3,7
YMR105C	PGM2	3,6
YDR154C		3,6
YJR104C	SOD1	3,5
YLR158C	ASP3-3	3,5
YLR438W	CAR2	3,4
YOL053C-A		3,4
YNL200C		3,4
YER026C	CHO1	3,1
YHL021C	FMP12	3,1
YHR137W	ARO9	3,1
YIR038C	GTT1	3,1
YDR155C	CPR1	3
YPR184W	GDB1	3
YJL151C	SNA3	3
YJR074W	MOG1	2,9
YMR090W		2,9
YML117W-A		2,9
YMR251W-A	HOR7	2,9
YHR033W		2,9
YGR209C	TRX2	2,9
YDR360W		2,9
YMR041C		2,9
YBR126C	TPS1	2,8
YNL208W		2,7
YNL274C		2,7
YLR356W		2,6
YPL154C	PEP4	2,6
YOL134C		2,6
YLR160C	ASP3-4	2,5
YBR173C	UMP1	2,5
YBR052C		2,5
YJL026W	RNR2	2,4
YNL003C	PET8	2,4
YJL217W		2,4
YER150W	SPI1	2,4
YAL012W	CYS3	2,4
YOL147C	PEX11	2,4
YMR276W	DSK2	2,4
YLR259C	HSP60	2,4
YGL077C	HNM1	2,3
YOR020C	HSP10	2,3
YOL110W	SHR5	2,3

Supplementary Table S1

YNL036W	NCE103	5,1	YMR096W	SNZ1	2,3
YIR038C	GTT1	3	YDR178W	SDH4	2,3
YGR279C	SCW4	3	YDR533C		2,3
YLL018C	DPS1	4	YHR113W		2,3
YMR173W	DDR48	3,5	YEL011W	GLC3	2,3
YMR116C	ASC1	3	YKL150W	MCR1	2,2
YKR066C	CCP1	3,6	YJL079C	PRY1	2,2
YNL134C		3,5	YOR382W	FIT2	2,2
YOR161C		3	YGL012W	ERG4	2,2
YKL019W	RAM2	3,5	YOR004W		2,2
YML070W	DAK1	2,8	YJR085C		2,2
YKL085W	MDH1	2,9	YCR061W		2,2
YGL123W	RPS2	3,9	YDR063W		2,2
YOR230W	WTM1	3,4	YEL060C	PRB1	2,2
YGR055W	MUP1	2,6	YBR287W		2,2
YLL045C	RPL8B	3,5	YAL039C	CYC3	2,2
YER178W	PDA1	4	YDR513W	TTR1	2,2
YML085C	TUB1	3,8	YCR044C	PER1	2,1
YJR139C	HOM6	3,5	YJR001W	AVT1	2,1
YBL099W	ATP1	3,5	YLR286C	CTS1	2,1
YOL129W	VPS68	2,5	YOR285W		2,1
YDL004W	ATP16	3,4	YBR214W	SDS24	2,1
YEL011W	GLC3	4	YDL014W	NOP1	2,1
YER062C	HOR2	3,8	YGL037C	PNC1	2,1
YBR074W		4,1	YIL112W	HOS4	2,1
YFR053C	HXK1	3,9	YDR134C		2,1
YML004C	GLO1	2,7	YPR183W	DPM1	2,1
YGR149W		2,8	YMR316W	DIA1	2,1
YER009W	NTF2	3,5	YDR095C		2,1
YGL185C		2,5	YLR202C		2,1
YGL026C	TRP5	2,8	YIL154C	IMP2'	2,1
YJL138C	TIF2	4,4	YMR120C	ADE17	2
YDR388W	RVS167	2,5	YMR071C	TVP18	2
YLR058C	SHM2	3,4	YLR043C	TRX1	2
YBR078W	ECM33	2,6	YCR067C	SED4	2
YJR028W		3,3	YLR391W-A		2
YDL046W	NPC2	2,5	YMR202W	ERG2	2
YJL026W	RNR2	3,9	YML078W	CPR3	2
YMR099C		3	YDR171W	HSP42	2
YDR368W	YPR1	2,8	YOL038W	PRE6	2
YER138C		5,6	YDR511W	ACN9	2
YML110C	COQ5	2,9	YOL079W		2
YNL274C		2,5	YOL165C	AAD15	2
YBR196C	PGI1	2,8	YLR155C	ASP3-1	2
YPL106C	SSE1	6	YPR053C		2
YPL135W	ISU1	3,3	YDR100W	TVP15	2
YBR162C	TOS1	3,5	YOR103C	OST2	2
YPR063C		2,8	YGL214W		0,5
YDR440W	DOT1	2,6	YGR102C		0,5
YLL044W		4,4	YPR023C	EAF3	0,5
YBR221C	PDB1	4,4	YCR087W		0,5
YGL011C	SCL1	3,3	YPL049C	DIG1	0,5
YDL204W	RTN2	2,4	YHR077C	NMD2	0,5
YML058W	SML1	2,3	YPL240C	HSP82	0,5
YNL071W	LAT1	3,7	YDR232W	HEM1	0,5
YHL021C		3,2	YOR359W	VTS1	0,5
YML012W	ERV25	3,2	YHR010W	RPL27A	0,5
YHR190W	ERG9	3	YMR233W		0,5

Supplementary Table S1

YDL066W	IDP1	2,9	YDL114W		0,5
YOR221C	MCT1	3,1	YBR034C	HMT1	0,5
YOL030W	GAS5	2,3	YOL059W	GPD2	0,5
YDR516C	EMI2	3,1	YKL148C	SDH1	0,5
YMR250W	GAD1	2,6	YBR189W	RPS9B	0,5
YHR092C	HXT4	2,3	YDR347W	MRP1	0,5
YPL262W	FUM1	2,7	YOL070C		0,5
YFR044C		2,5	YHR097C		0,5
YLL039C	UBI4	2,7	YER086W	ILV1	0,5
YML048W	GSF2	2,3	YMR278W		0,5
YHR017W	YSC83	2,3	YGR103W	NOP7	0,5
YLR354C	TAL1	2,3	YPL051W	ARL3	0,5
YGR204W	ADE3	2,9	YDR174W	HMO1	0,5
YEL027W	CUP5	2,8	YKL019W	RAM2	0,5
YDR099W	BMH2	3,5	YER036C		0,5
YDR483W	KRE2	2,8	YBR154C	RPB5	0,5
YHR071W	PCL5	2,8	YDR277C	MTH1	0,5
YGL202W	ARO8	3	YLR459W	CDC91	0,5
YOR065W	CYT1	2,5	YJR145C	RPS4A	0,5
YBL030C	PET9	2,8	YBR288C	APM3	0,5
YLL024C	SSA2	3,5	YGL123W	RPS2	0,5
YDR342C	HXT7	2,3	YJR007W	SUI2	0,5
YBR149W	ARA1	2,5	YBL072C	RPS8A	0,5
YMR316W	DIA1	2,5	YDR341C		0,5
YOR374W	ALD4	3,3	YHR160C	PEX18	0,5
YDR538W	PAD1	2,5	YDL161W	ENT1	0,5
YGR268C	HUA1	2,5	YGL148W	ARO2	0,5
YKR059W	TIF1	2,8	YDR033W	MRH1	0,5
YDR055W	PST1	2,1	YHR043C	DOG2	0,5
YBR139W		2,5	YLR256W	HAP1	0,5
YGL191W	COX13	2,3	YPL081W	RPS9A	0,5
YHR207C	SET5	3,3	YGR159C	NSR1	0,5
YBR056W		3	YMR083W	ADH3	0,5
YPR196W		2,7	YJL148W	RPA34	0,5
YBR035C	PDX3	2,4	YCL059C	KRR1	0,5
YLR150W	STM1	2,7	YDR245W	MNN10	0,5
YBR288C	APM3	2,9	YGR086C	PIL1	0,5
YOR153W	PDR5	2,2	YER165W	PAB1	0,5
YJR027W		5,3	YML026C	RPS18B	0,5
YOL053C-A		2,2	YOR078W	BUD21	0,5
YNR001C	CIT1	2,8	YNL016W	PUB1	0,5
YDL237W		2,5	YOL041C	NOP12	0,5
YFL039C	ACT1	2,2	YMR236W	TAF9	0,5
YER043C	SAH1	2,5	YER081W	SER3	0,5
YER102W	RPS8B	2,9	YLR201C	FMP53	0,5
YDR345C	HXT3	2,1	YLR300W	EXG1	0,5
YER090W	TRP2	3	YGR078C	PAC10	0,5
YKL100C		2	YLR196W	PWP1	0,5
YGR106C		2,4	YGR137W		0,5
YER160C		4,3	YNL030W	HHF2	0,5
YHR042W	NCP1	2,1	YOR047C	STD1	0,5
YDR343C	HXT6	2,2	YIL074C	SER33	0,5
YDL139C	SCM3	2,1	YHL003C	LAG1	0,5
YMR163C		0,5	YER056C-A	RPL34A	0,5
YPL254W	HFI1	0,5	YHR042W	NCP1	0,5
YKL099C	UTP11	0,5	YKL211C	TRP3	0,5
YER019W	ISC1	0,4	YLR289W	GUF1	0,5
YDL121C		0,5	YMR058W	FET3	0,5

YER107C	GLE2	0,5	YLR332W	MID2	0,5
YOR392W		0,5	YDR518W	EUG1	0,5
YPL059W	GRX5	0,5	YLR339C		0,5
YLR297W		0,4	YKL185W	ASH1	0,5
YOR339C	UBC11	0,5	YHR020W		0,5
YOR041C		0,3	YER055C	HIS1	0,5
YPL095C		0,5	YFR031C-A	RPL2A	0,5
YDR424C	DYN2	0,4	YGR285C	ZUO1	0,5
YDR193W		0,4	YLR330W	CHS5	0,5
YJR074W	MOG1	0,5	YLR150W	STM1	0,5
YOR224C	RPB8	0,5	YLR143W		0,5
YKL222C		0,5	YPR080W	TEF1	0,5
YCR013C		0,4	YHR030C	SLT2	0,5
YPL192C	PRM3	0,5	YPR181C	SEC23	0,5
YKL002W	DID4	0,5	YDR046C	BAP3	0,5
YPR082C	DIB1	0,4	YPR010C	RPA135	0,5
YPR153W		0,4	YOR145C	PNO1	0,5
YDR511W	ACN9	0,4	YKL176C	LST4	0,5
YPR200C	ARR2	0,3	YGR034W	RPL26B	0,5
YPR123C		0,4	YGL181W	GTS1	0,5
YPR017C	DSS4	0,4	YOL148C	SPT20	0,5
YOL012C	HTZ1	0,5	YMR194W	RPL36A	0,5
YDR053W		0,3	YGR268C	HUA1	0,5
YER105C	NUP157	0,5	YHL001W	RPL14B	0,5
YDR357C		0,5	YDL133W		0,5
YPR100W	MRPL51	0,4	YBR011C	IPP1	0,5
YDR433W		0,5	YIL053W	RHR2	0,5
YOR200W		0,4	YIL078W	THS1	0,5
YPR168W	NUT2	0,5	YKL180W	RPL17A	0,5
YCL013W		0,4	YMR121C	RPL15B	0,5
YDL123W	SNA4	0,5	YOR051C		0,5
YHR165C	PRP8	0,5	YFL005W	SEC4	0,5
YOR382W	FIT2	0,4	YDR091C	RLI1	0,5
YOL165C	AAD15	0,4	YPL219W	PCL8	0,5
YCR046C	IMG1	0,5	YAL003W	EFB1	0,5
YDR100W	TVP15	0,5	YEL054C	RPL12A	0,5
YOL079W		0,4	YAL005C	SSA1	0,5
YER137C		0,5	YDR450W	RPS18A	0,5
YOR286W		0,5	YKR094C	RPL40B	0,5
YCRX12W		0,5	YBR121C	GRS1	0,5
YIL121W	QDR2	0,5	YDL191W	RPL35A	0,5
YNL329C	PEX6	0,4	YDR447C	RPS17B	0,5
YGL077C	HNM1	0,5	YOR168W	GLN4	0,5
YHR021C	RPS27B	0,5	YDR068W	DOS2	0,5
YOR103C	OST2	0,3	YNL062C	GCD10	0,5
YPR193C	HPA2	0,4	YBL068W	PRS4	0,5
YOR055W		0,4	YHR182C-A		0,5
YPL200W	CSM4	0,3	YMR108W	ILV2	0,5
YDR366C		0,3	YNL112W	DBP2	0,5
YPL163C	SVS1	0,5	YGL009C	LEU1	0,5
YNL170W		0,5	YER183C	FAU1	0,5
YBL002W	HTB2	0,5	YDR259C	YAP6	0,5
YHL025W	SNF6	0,5	YDR471W	RPL27B	0,5
YOR012W		0,4	YBR048W	RPS11B	0,5
YBL083C		0,5	YOR096W	RPS7A	0,5
YPL225W		0,5	YGR085C	RPL11B	0,4
YPR188C	MLC2	0,5	YGR082W	TOM20	0,4
YOR052C		0,4	YML024W	RPS17A	0,4

Supplementary Table S1

YGR184C	UBR1	0,5	YNL178W	RPS3	0,4
YPR064W		0,4	YGL147C	RPL9A	0,4
YOR226C	ISU2	0,5	YBR084C-A	RPL19A	0,4
YDR445C		0,5	YGL076C	RPL7A	0,4
YPL276W	FDH2	0,3	YOL109W	ZEO1	0,4
YBR296C	PHO89	0,4	YPL281C	ERR2	0,4
YPL238C		0,3	YLR029C	RPL15A	0,4
YEL077C		0,5	YDL075W	RPL31A	0,4
YDR344C		0,5	YLL018C	DPS1	0,4
YGL255W	ZRT1	0,4	YPL007C	TFC8	0,4
YDR442W		0,5	YML042W	CAT2	0,4
YPR130C		0,3	YDR075W	PPH3	0,4
YDR437W		0,4	YPL079W	RPL21B	0,4
YOR325W		0,5	YMR046C		0,4
YBR291C	CTP1	0,3	YJL200C		0,4
YPR050C		0,3	YOR063W	RPL3	0,4
YDR060W	MAK21	0,5	YKL081W	TEF4	0,4
YIR035C		0,4	YMR309C	NIP1	0,4
YOR263C		0,3	YIL133C	RPL16A	0,4
YOL108C	INO4	0,3	YLR448W	RPL6B	0,4
YOR218C		0,3	YOL120C	RPL18A	0,4
YDR012W	RPL4B	0,5	YPR102C	RPL11A	0,4
YDR544C		0,5	YNL067W	RPL9B	0,4
YOR183W	FYV12	0,3	YJR026W		0,4
YDR073W	SNF11	0,5	YNL050C		0,4
YLR186W	EMG1	0,3	YIL069C	RPS24B	0,4
YDR095C		0,4	YBR115C	LYS2	0,4
YJR024C		0,4	YJR045C	SSC1	0,4
YOR139C		0,3	YMR217W	GUA1	0,4
YDR462W	MRPL28	0,5	YLR175W	CBF5	0,4
YMR103C		0,5	YOR133W	EFT1	0,4
YJR113C	RSM7	0,4	YFL063W		0,4
YOR333C	SWF5	0,4	YDR025W	RPS11A	0,4
YDR063W		0,4	YNL042W	BOP3	0,4
YOR044W		0,3	YPL190C	NAB3	0,4
YLR198C		0,5	YML056C	IMD4	0,4
YDR156W	RPA14	0,4	YKR059W	TIF1	0,4
YNL300W		0,5	YNL069C	RPL16B	0,4
YOR222W	ODC2	0,4	YMR153W	NUP53	0,4
YDR526C		0,3	YDR367W		0,4
YOR257W	CDC31	0,5	YDL184C	RPL41A	0,4
YOR013W		0,3	YNL104C	LEU4	0,4
YPL111W	CAR1	0,5	YPL090C	RPS6A	0,4
YOR169C		0,3	YGR240C	PFK1	0,4
YMR308C	PSE1	0,4	YJL189W	RPL39	0,4
YOR252W		0,3	YBL027W	RPL19B	0,4
YDR224C	HTB1	0,5	YIL052C	RPL34B	0,4
YDL125C	HNT1	0,3	YNL096C	RPS7B	0,4
YGR085C	RPL11B	0,4	YML073C	RPL6A	0,4
YBL077W		0,4	YBR092C	PHO3	0,4
YFL007W	BLM3	0,3	YDR382W	RPP2B	0,4
YDR225W	HTA1	0,3	YBR191W	RPL21A	0,4
YMR241W	YHM2	0,3	YDL082W	RPL13A	0,4
YDL208W	NHP2	0,4	YDR318W	MCM21	0,4
YLR029C	RPL15A	0,3	YGR148C	RPL24B	0,4
YER053C		0,3	YGR118W	RPS23A	0,4
YOR063W	RPL3	0,3	YMR235C	RNA1	0,4
YHR030C	SLT2	0,4	YPR065W	ROX1	0,4

Supplementary Table S1

YPL249C	GYP5	0,3
YMR121C	RPL15B	0,3
YEL033W		0,3
YJL129C	TRK1	0,3
YBL003C	HTA2	0,3
YDR093W	DNF2	0,2
YNL112W	DBP2	0,2
YDL171C	GLT1	0,2
YBR031W	RPL4A	0,2
YGR159C	NSR1	0,3
YNL178W	RPS3	0,1

YER102W	RPS8B	0,4
YLL048C	YBT1	0,4
YKL077W		0,4
YMR051C		0,4
YMR231W	PEP5	0,4
YEL053W-A		0,4
YJL034W	KAR2	0,4
YMR242C	RPL20A	0,4
YPL160W	CDC60	0,4
YAR010C		0,4
YDR481C	PHO8	0,4
YDL218W		0,4
YOL097C	WRS1	0,4
YLR197W	SIK1	0,4
YPL116W	HOS3	0,4
YDR234W	LYS4	0,4
YGR119C	NUP57	0,4
YHR219W		0,4
YEL036C	ANP1	0,4
YOR393W	ERR1	0,4
YHR135C	YCK1	0,4
YBR054W	YRO2	0,4
YOR351C	MEK1	0,4
YGR214W	RPS0A	0,4
YNL251C	NRD1	0,4
YML010W-A		0,4
YDR417C		0,4
YLR249W	YEF3	0,4
YGL245W		0,4
YPL009C		0,4
YJR028W		0,4
YDR505C	PSP1	0,4
YOR312C	RPL20B	0,4
YPL106C	SSE1	0,4
YOR195W	SLK19	0,4
YDR346C	SVF1	0,4
YGR094W	VAS1	0,4
YJR121W	ATP2	0,3
YDL136W	RPL35B	0,3
YGL206C	CHC1	0,3
YBR181C	RPS6B	0,3
YGL103W	RPL28	0,3
YHR094C	HXT1	0,3
YOR293W	RPS10A	0,3
YDR023W	SES1	0,3
YML045W		0,3
YJR029W		0,3
YML040W		0,3
YLR075W	RPL10	0,3
YDL052C	SLC1	0,3
YLL044W		0,3
YLR048W	RPS0B	0,3
YMR142C	RPL13B	0,3
YHL033C	RPL8A	0,3
YDR385W	EFT2	0,3
YDR035W	ARO3	0,3
YPL131W	RPL5	0,3
YPR033C	HTS1	0,3

YPR145W	ASN1	0,3
YNL223W	ATG4	0,3
YGL026C	TRP5	0,3
YLL045C	RPL8B	0,3
YNL190W		0,3
YPL198W	RPL7B	0,3
YER131W	RPS26B	0,3
YIL131C	FKH1	0,3
YJL138C	TIF2	0,3
YIL094C	LYS12	0,3
YNL031C	HHT2	0,3
YOR309C		0,3
YHL015W	RPS20	0,3
YOR335C	ALA1	0,3
YHR064C	SSZ1	0,3
YJR027W		0,3
YCR053W	THR4	0,2
YBR010W	HHT1	0,2
YBR009C	HHF1	0,2
YLR413W		0,2
YBL002W	HTB2	0,2
YML063W	RPS1B	0,2
YGR229C	SMI1	0,2
YNL209W	SSB2	0,2
YCL030C	HIS4	0,2
YER138C		0,2
YPR074C	TKL1	0,2
YGL008C	PMA1	0,2
YER160C		0,2
YBL003C	HTA2	0,2
YGR069W		0,2
YNL230C	ELA1	0,1
YGL055W	OLE1	0,1
YDR116C	MRPL1	0,1
