# Supplementary material for: Genome-Wide Analysis of Factors Affecting Transcription Elongation and DNA Repair: A New Role for PAF and Ccr4-Not in Transcription-Coupled Repair
Source: PLoS Genet. 2009 Feb 6;5(2):e1000364. doi: 10.1371/journal.pgen.1000364 (PMC2629578; doi:10.1371/journal.pgen.1000364)
Supplement: Table S2 — Analysis of mutant strains leading to 4-NQO sensitivity. Growth was monitored after 48, 72, and 120 hours in 4-NQO-containing media (150 ng/ml) and the sensitive strains classified into three groups. Group A contains 189 strains whose sensitivity to 4-NQO is observed from early on (48 h) and is maintained over the course of the experiment (up to 120 h for group A1, and up to 72 h for group A2), group B contains 315 strains whose sensitivity was observed only at early time points (48 h), and group C contains 100 slow-growing strains which showed 4-NQO sensitivity only at later time points (from 72 h on and up to 120 h). The strains of group B were not considered as significantly inhibited by 4-NQO and excluded from further analyses. Values between 60% and 80% and above 80% inhibition are highlighted (orange and red, respectively). (0.07 MB PDF) [file pgen.1000364.s003.pdf]

Supplementary Table S2

Group A1:

ORF	Name	%INH 4NQO 48h	%INH 4NQO 72h	%INH 4NQO 120h
YLR032W	RAD5	90,59	90,74	93,53
YMR201C	RAD14	92,64	93,04	92,94
YNL073W	MSK1	88,78	92,83	92,75
YMR190C	SGS1	92,67	92,88	92,67
YHR051W	COX6	82,44	92,33	92,33
YNL081C	SWS2	86,55	92,44	92,33
YPL022W	RAD1	92,20	92,33	92,33
YLR295C	ATP14	91,87	92,37	92,27
YLR235C		92,23	92,25	92,25
YJL124C	LSM1	92,06	92,23	92,23
YPL024W	NCE4	92,12	92,05	92,16
YER050C	RSM18	89,78	90,79	92,13
YPL194W	DDC1	92,12	92,38	92,08
YMR151W	YIM2	92,02	92,08	92,08
YML096W		91,32	91,38	92,05
YOR231W	MKK1	92,08	92,05	92,05
YBR101C	FES1	91,74	91,80	91,99
YLR234W	TOP3	91,88	91,96	91,96
YDL005C	MED2	91,64	91,79	91,89
YLR038C	COX12	90,64	91,00	91,76
YEL037C	RAD23	91,81	91,36	91,75
YML129C	COX14	91,02	91,27	91,68
YPR124W	CTR1	86,74	91,58	91,67
YMR084W		84,18	90,99	91,62
YLR320W	MMS22	91,49	91,57	91,57
YML095C	RAD10	90,85	90,94	91,53
YMR097C	MTG1	90,67	90,89	91,53
YEL044W	IES6	91,05	90,83	91,52
YPL078C		90,83	91,51	91,51
YKR085C	MRPL20	88,47	92,12	91,50
YER095W	RAD51	89,92	89,96	91,50
YLR182W	SWI6	91,58	91,60	91,49
YOR065W	CYT1	81,40	91,47	91,47
YGL058W	RAD6	90,23	90,56	91,44
YBR114W	RAD16	91,05	91,26	91,44
YGL025C	PGD1	90,81	91,24	91,44
YOR201C	PET56	91,14	91,39	91,39
YFL036W	RPO41	87,65	90,47	91,38
YGL024W		90,33	90,99	91,38
YDL116W	NUP84	90,84	91,23	91,33
YOL051W	GAL11	91,06	91,27	91,27
YJL140W	RPB4	91,18	91,65	91,22
YML007W	YAP1	90,81	91,11	91,21
YNL139C	RLR1	91,21	91,07	91,17
YMR158W	MRPS8	89,84	89,99	91,16
YMR072W	ABF2	81,87	90,35	91,16
YLR226W	BUR2	90,40	91,02	91,13
YML011C		91,92	92,15	91,10

YJL075C		90,60	91,20	90,99
YPR047W	MSF1	84,38	90,98	90,98
YPR116W		84,06	90,77	90,97
YLR240W	VPS34	90,86	90,94	90,94
YLR399C	BDF1	90,68	90,78	90,90
YBR094W		90,68	90,88	90,88
YDR337W	MRPS28	87,16	90,78	90,87
YDR237W	MRPL7	90,37	90,85	90,85
YML032C	RAD52	90,66	90,95	90,84
YCR053W	THR4	90,33	90,94	90,84
YGL163C	RAD54	89,81	90,32	90,74
YDR264C	AKR1	90,50	90,62	90,72
YLR244C	MAP1	90,81	90,94	90,71
YGL070C	RPB9	80,22	90,52	90,63
YDR322W	MRPL35	88,61	90,60	90,60
YDL160C	DHH1	90,20	90,50	90,59
YKL054C	DEF1	90,53	90,69	90,58
YER103W	SSA4	90,12	90,53	90,53
YDR138W	HPR1	88,85	90,38	90,48
YBR037C	SCO1	90,11	90,48	90,48
YBR251W	MRPS5	82,42	90,36	90,46
YER162C	RAD4	90,11	90,34	90,43
YDL057W		89,73	90,32	90,42
YDR296W	MHR1	87,65	90,18	90,39
YPL132W	COX11	89,49	90,70	90,38
YDR004W	RAD57	89,92	90,26	90,35
YAL021C		90,13	90,21	90,31
YCR003W	MRPL32	88,70	90,19	90,29
YDL167C	NRP1	89,17	90,36	90,26
YOL148C	SPT20	88,44	90,62	90,23
YMR198W	CIK1	91,94	91,91	90,17
YDL067C	COX9	89,61	90,06	90,17
YGL167C	PMR1	90,23	90,59	90,14
YDR230W		89,50	90,04	90,14
YHR091C	MSR1	86,13	92,12	90,05
YCR066W	RAD18	90,72	90,69	90,04
YDR268W	MSW1	83,55	89,93	90,03
YDR283C	GCN2	89,27	89,81	89,91
YKL194C	MST1	84,68	90,32	89,89
YBL045C	COR1	84,18	90,08	89,88
YHR168W		82,41	88,92	89,87
YDL135C	RDI1	87,02	89,85	89,85
YIR021W	MRS1	89,40	89,73	89,84
YJL115W	ASF1	89,80	89,98	89,76
YDR028C	REG1	89,48	89,62	89,73
YER169W	RPH1	81,44	89,92	89,71
YGR258C	RAD2	89,41	90,09	89,61
YNL284C	MRPL10	88,78	89,99	89,59
YJR052W	RAD7	90,04	89,93	89,55
YOR033C	EXO1	85,04	89,39	89,52
YPL045W	VPS16	89,38	89,51	89,51
YDL101C	DUN1	89,19	89,60	89,49
YGR112W	SHY1	83,03	89,66	89,46

YBL093C	ROX3	88,67	89,38	89,38
YDR369C	XRS2	88,44	89,06	89,06
YGL038C	OCH1	86,62	88,39	89,03
YBR098W	MMS4	90,37	90,55	89,17
YNL250W	RAD50	90,74	90,88	88,93
YNL059C	ARP5	87,90	88,93	88,84
YCR046C	IMG1	85,83	88,90	88,69
YAL016W	TPD3	86,62	87,09	87,09
YOR330C	MIP1	86,00	86,72	86,23
YJL127C	SPT10	90,15	90,06	85,46
YDL069C	CBS1	89,84	90,24	84,31
YGR104C	SRB5	89,56	89,78	82,75
YGR062C	COX18	89,86	90,43	78,09
YDR011W	SNQ2	90,63	90,75	72,16
YIL139C	REV7	91,19	91,20	71,95

Group A2:

ORF	Name	%INH 4NQO 48h	%INH 4NQO 72h	%INH 4NQO 120h
YGL175C	SAE2	92,16	93,12	9,02
YNL236W	SIN4	92,05	91,86	2,22
YBR099C		90,77	91,73	1,12
YOL076W	MDM20	91,39	91,69	-11,31
YPL008W	CHL1	92,50	91,68	4,88
YNL064C	YDJ1	92,00	91,53	17,59
YFR010W	UBP6	91,89	91,42	3,21
YDR217C	RAD9	89,87	91,28	18,96
YDR405W	MRP20	90,69	91,19	-0,20
YIR002C	MPH1	91,13	91,02	24,97
YJL092W	HPR5	91,30	90,98	17,01
YJR043C	POL32	91,61	90,70	4,11
YGR063C	SPT4	90,60	90,62	-1,40
YKL212W	SAC1	91,28	90,61	2,27
YDR386W	MUS81	89,48	90,58	1,59
YLR218C		91,57	90,50	14,62
YDL059C	RAD59	89,94	90,49	-7,43
YPR120C	CLB5	91,38	90,38	3,95
YPL046C	ELC1	92,55	90,34	6,26
YNL147W	LSM7	90,06	90,30	-7,13
YKL113C	RAD27	90,63	90,29	1,18
YPR131C	NAT3	91,29	90,10	-1,06
YOR346W	REV1	91,98	89,92	25,77
YDL077C	VAM6	90,33	89,91	6,52
YOR036W	PEP12	91,44	89,62	1,45
YLR418C	CDC73	90,32	89,61	18,99
YPR141C	KAR3	91,30	89,46	7,29
YHR041C	SRB2	90,68	89,40	-0,41
YLR055C	SPT8	91,46	89,34	53,71
YEL051W	VMA8	91,32	89,21	0,92
YBR133C	HSL7	91,73	89,12	-3,33
YDL115C		90,50	88,96	0,20

Supplementary Table S2

YOR141C	ARP8	92,10	88,94	6,08
YNL199C	GCR2	89,95	88,82	2,37
YBR097W	VPS15	88,87	88,51	62,30
YDR076W	RAD55	89,43	88,35	-4,34
YOR331C		90,25	88,24	3,30
YOL072W	THP1	91,16	87,73	66,56
YLL043W	FPS1	91,99	87,49	-2,56
YLR006C	SSK1	91,36	86,86	-5,56
YPL172C	COX10	91,34	86,76	6,38
YKL139W	CTK1	91,09	86,73	-0,69
YLR376C		91,72	86,48	18,99
YMR091C	NPL6	89,51	86,02	10,07
YPR164W	MMS1	91,48	85,71	-3,11
YPL268W	PLC1	90,84	85,65	5,72
YPL055C	LGE1	91,35	85,58	-1,74
YLR382C	NAM2	90,91	85,25	6,78
YPL013C	MRPS16	91,72	84,80	27,67
YCL007C	CWH36	89,35	84,31	-6,64
YLL002W	RTT109	90,75	83,66	-2,53
YBL058W	SHP1	88,28	83,25	0,76
YLR358C		90,34	83,23	7,39
YPL234C	TFP3	91,43	83,03	3,02
YGL168W	HUR1	89,85	82,96	0,59
YJL189W	RPL39	90,69	82,66	-3,40
YML010W-A	SPT5	90,97	82,15	-0,74
YJR139C	HOM6	90,63	82,10	1,64
YBR081C	SPT7	89,46	81,72	-1,96
YJR018W		90,52	81,53	16,36
YJL184W	GON7	89,76	81,30	-2,16
YOR080W	DIA2	90,64	80,92	6,52
YPL129W	TAF14	90,39	80,77	-7,65
YMR231W	PEP5	88,47	80,23	68,99
YER110C	KAP123	87,87	79,98	0,33
YPR100W	MRPL51	88,61	77,60	-1,59
YMR064W	AEP1	85,58	76,68	-0,41
YJL006C	CTK2	86,17	75,31	8,09
YOL095C	HMI1	84,68	74,58	36,93
YBR112C	CYC8	82,45	73,90	-3,81
YML112W	CTK3	82,20	73,89	-3,38
YEL024W	RIP1	82,28	73,08	-12,56
YLR233C	EST1	82,29	72,39	-6,40

Group B:

ORF	Name	%INH 4NQO 48h	%INH 4NQO 72h	%INH 4NQO 120h
YOR368W	RAD17	92,39	54,28	-2,06
YOR332W	VMA4	91,98	10,76	-0,43
YMR031W-A		91,74	44,08	-1,92
YOL012C	HTZ1	91,48	43,00	-4,15
YMR073C		91,36	6,23	-12,07
YLR268W	SEC22	91,32	3,43	0,00

Supplementary Table S2

YNL235C		91,25	0,40	-1,01
YOL004W	SIN3	91,23	2,54	-0,20
YMR224C	MRE11	91,20	-8,49	-8,90
YPR119W	CLB2	91,20	0,00	-3,30
YBR159W		91,17	-1,44	-5,17
YER016W	BIM1	91,15	15,35	2,70
YEL059W		91,10	9,72	3,40
YOL081W	IRA2	91,10	19,58	9,74
YNL107W	YAF9	91,10	25,37	-0,88
YJL047C	RTT101	91,09	12,93	8,94
YKR082W	NUP133	91,08	64,80	-0,39
YNL171C		91,07	14,71	0,10
YGR064W		91,05	7,04	4,17
YJR059W	PTK2	90,99	9,75	3,59
YMR202W	ERG2	90,98	28,11	-1,32
YOR026W	BUB3	90,80	0,91	-3,15
YHR060W	VMA22	90,78	23,02	5,24
YKR072C	SIS2	90,78	1,25	-2,40
YJR090C	GRR1	90,77	67,47	-2,23
YNL133C	FYV6	90,70	56,96	3,87
YPL173W	MRPL40	90,68	1,62	-5,17
YGL087C	MMS2	90,58	21,90	1,55
YHL006C	SHU1	90,54	6,09	5,00
YLR261C	VPS63	90,54	-6,99	-8,12
YML001W	YPT7	90,49	9,92	9,53
YLR052W	IES3	90,46	24,90	-3,77
YIR026C	YVH1	90,45	47,66	-0,20
YMR083W	ADH3	90,41	0,65	-3,27
YMR307W	GAS1	90,36	1,98	-3,26
YGR020C	VMA7	90,32	2,62	-2,30
YNL198C		90,30	5,21	-1,10
YKL119C	VPH2	90,26	60,67	-0,49
YOR039W	CKB2	90,26	37,93	-1,76
YER173W	RAD24	90,26	4,37	-1,14
YBR093C	PHO5	90,25	10,75	7,47
YLL041C	SDH2	90,22	-0,30	-1,80
YDR378C	LSM6	90,20	1,43	0,86
YNL248C	RPA49	90,18	0,49	-4,85
YKL006W	RPL14A	90,16	28,04	-5,92
YMR034C		90,13	1,35	-4,06
YGL246C	RAI1	90,10	49,73	5,13
YNL298W	CLA4	90,05	0,42	-1,48
YIL128W	MET18	90,03	49,46	4,87
YLR318W	EST2	90,02	-10,16	-14,29
YGL244W	RTF1	90,02	23,96	3,42
YFR001W	LOC1	90,02	3,41	1,95
YPL051W	ARL3	90,01	-1,29	-2,87
YHR025W	THR1	90,00	46,19	-2,77
YJL176C	SWI3	90,00	54,51	4,40
YBR244W	GPX2	89,91	-1,34	-11,28
YDR162C	NBP2	89,89	5,64	4,55
YOR007C	SGT2	89,88	-1,76	-10,55
YBR231C		89,87	4,24	2,50

Supplementary Table S2

YDR207C	UME6	89,87	13,06	5,82
YOR306C	MCH5	89,86	13,37	-2,84
YPR159W	KRE6	89,82	3,41	-0,47
YGL012W	ERG4	89,79	-17,25	-7,38
YLR372W	SUR4	89,79	0,35	-3,86
YDL185W	TFP1	89,79	-4,89	-4,08
YNL307C	MCK1	89,74	-0,82	-8,56
YOR221C	MCT1	89,73	17,81	-2,30
YBR127C	VMA2	89,72	-0,65	-5,29
YKR028W	SAP190	89,71	3,33	-1,27
YDR174W	HMO1	89,70	38,40	-8,02
YPL167C	REV3	89,68	-2,31	-3,56
YDR173C	ARG82	89,67	14,73	4,41
YKL048C	ELM1	89,62	14,57	8,32
YMR125W	STO1	89,62	18,96	-1,80
YPR036W	VMA13	89,57	-0,99	-6,26
YOR018W	ROD1	89,56	-4,84	-6,26
YDL074C	BRE1	89,54	37,83	-19,27
YDR159W	SAC3	89,49	65,28	12,44
YDR092W	UBC13	89,49	16,17	7,33
YLR319C	BUD6	89,44	1,25	-2,11
YOR035C	SHE4	89,43	1,81	-1,71
YDR432W	NPL3	89,43	56,09	4,67
YOL006C	TOP1	89,43	-3,64	-2,73
YHR154W	RTT107	89,42	3,80	2,63
YPR163C	TIF3	89,42	1,42	-11,44
YIL116W	HIS5	89,39	0,48	-2,58
YHR021C	RPS27B	89,38	8,43	3,31
YHR191C	CTF8	89,38	11,23	4,03
YMR272C	SCS7	89,34	6,29	5,55
YGL088W		89,27	-0,46	-2,40
YKL121W		89,23	-3,16	-4,24
YBR103W	SIF2	89,21	3,83	-2,20
YNL229C	URE2	89,21	33,72	-1,92
YBR145W	ADH5	89,16	3,30	-3,11
YML102C-A		89,16	0,19	-2,41
YMR216C	SKY1	89,15	2,57	-0,57
YGR252W	GCN5	89,13	-0,63	-12,70
YGL151W	NUT1	89,13	-12,21	-17,58
YCR077C	PAT1	89,12	9,76	4,74
YGR105W	VMA21	89,03	-5,72	-6,46
YDR254W	CHL4	89,02	11,01	8,94
YBR268W	MRPL37	88,98	-41,17	-9,68
YOR295W	UAF30	88,94	2,59	-1,69
YEL027W	CUP5	88,93	0,61	-1,32
YER014C-A	BUD25	88,92	-0,52	-3,41
YDR364C	CDC40	88,90	52,95	4,47
YOR017W	PET127	88,83	-5,71	-12,14
YCL060C	KRR1	88,83	7,12	4,51
YDL172C		88,76	5,28	2,25
YJL064W		88,75	7,08	4,46
YLR373C	VID22	88,72	13,64	-12,14
YAR002W	NUP60	88,70	-4,18	-5,38

Supplementary Table S2

YAL047C		88,69	-4,42	-8,39
YCL016C	DCC1	88,66	12,09	10,82
YDL081C	RPP1A	88,64	4,99	-0,49
YDL117W	CYK3	88,61	10,31	11,01
YOR014W	RTS1	88,61	-1,68	-3,85
YLR398C	SKI2	88,60	-1,34	-6,30
YOR345C		88,59	-3,07	-3,59
YMR038C	LYS7	88,55	1,33	-2,57
YDL075W	RPL31A	88,52	2,15	1,23
YGL124C	MON1	88,52	-0,48	-0,68
YPR139C	VPS66	88,52	16,67	5,21
YDR290W		88,46	-8,43	-8,22
YIL040W		88,37	14,84	12,01
YPR194C	OPT2	88,35	4,70	1,60
YBR116C		88,33	8,16	-1,96
YOR078W	BUD21	88,32	0,21	-5,31
YML111W	BUL2	88,28	2,31	0,48
YKL118W		88,27	-9,76	-11,95
YDL006W	PTC1	88,19	55,73	0,60
YGR240C	PFK1	88,18	10,25	0,47
YLR056W	ERG3	88,09	5,08	-1,37
YML102W	CAC2	88,09	1,93	-0,39
YML097C	VPS9	88,01	9,62	-1,51
YLR039C	RIC1	88,00	-3,94	-6,29
YDR017C	KCS1	87,99	10,86	2,56
YDR158W	HOM2	87,95	6,65	0,81
YDR195W	REF2	87,92	19,50	4,10
YLR357W	RSC2	87,91	-0,21	-7,29
YOL017W	ESC8	87,90	0,29	-5,00
YLR322W	VPS65	87,87	42,28	-2,28
YMR022W	QRI8	87,86	-2,55	-4,02
YOR025W	HST3	87,80	-1,76	-15,81
YKR024C	DBP7	87,79	40,16	-8,30
YGR208W	SER2	87,78	5,41	4,38
YDL013W	HEX3	87,77	10,15	5,79
YLR447C	VMA6	87,64	-4,91	-4,35
YJR118C	ILM1	87,64	2,70	-6,58
YBL080C	PET112	87,54	8,66	6,76
YDL232W	OST4	87,51	44,35	2,11
YGL212W	VAM7	87,42	-1,64	3,08
YLR062C	BUD28	87,37	0,41	-1,32
YLR015W	BRE2	87,37	7,20	2,65
YJR073C	OPI3	87,24	6,51	3,25
YPR135W	CTF4	87,19	-7,49	-9,23
YMR032W	HOF1	87,17	-1,18	-4,31
YLR192C	HCR1	87,14	2,36	-0,66
YBR191W	RPL21A	87,08	1,25	-3,85
YEL013W	VAC8	87,02	9,20	0,49
YDR216W	ADR1	86,98	0,95	-1,90
YPR057W	BRR1	86,92	-3,89	-7,08
YBR266C		86,80	2,75	0,37
YMR304W	UBP15	86,76	-1,55	-3,30
YBR128C	ATG14	86,75	3,59	-1,26

Supplementary Table S2

YNL220W	ADE12	86,73	38,22	21,27
YCR002C	CDC10	86,67	3,40	1,65
YGL020C	MDM39	86,61	7,01	1,38
YOR223W		86,59	-3,59	-6,08
YLR412W		86,58	-5,39	-10,10
YGL218W		86,55	2,84	-7,93
YDL100C	ARR4	86,50	4,31	2,49
YDL047W	SIT4	86,46	-2,01	-2,54
YGL007W		86,44	3,27	0,53
YDR245W	MNN10	86,43	-0,89	-2,57
YFR019W	FAB1	86,35	-2,45	-2,36
YLR025W	SNF7	86,32	-1,01	2,81
YLR102C	APC9	86,30	2,27	-1,38
YKL126W	YPK1	86,28	-1,51	-2,83
YIL132C	CSM2	86,28	9,02	4,64
YBR267W		86,24	3,53	2,36
YMR116C	ASC1	86,19	4,57	-1,89
YGL136C	MRM2	86,15	-7,61	-6,90
YML008C	ERG6	86,13	-7,53	-7,85
YPR045C		86,11	7,49	-0,64
YJL036W	SNX4	86,09	2,66	1,19
YJL052W	TDH1	85,91	3,89	1,07
YML063W	RPS1B	85,85	-2,95	-7,73
YLR396C	VPS33	85,85	-90,93	-91,11
YDR392W	SPT3	85,84	3,11	-1,35
YCR028C	FEN2	85,76	25,79	8,49
YBR131W	CCZ1	85,74	-58,44	-64,69
YEL042W	GDA1	85,74	0,69	1,09
YOR290C	SNF2	85,74	-0,30	-3,32
YGL042C		85,67	0,89	0,40
YDR027C	VPS54	85,67	-4,01	-7,01
YMR166C		85,57	-0,19	-3,37
YOR038C	HIR2	85,56	-0,32	-5,57
YJR116W		85,56	-3,13	-23,66
YMR167W	MLH1	85,50	4,17	-0,28
YOR040W	GLO4	85,49	-1,58	-3,89
YMR143W	RPS16A	85,47	2,33	-2,04
YKL080W	VMA5	85,40	1,02	20,15
YLR061W	RPL22A	85,35	3,09	-3,09
YIL148W	RPL40A	85,34	5,68	6,65
YOR032C	HMS1	85,27	-2,77	-7,63
YKL101W	HSL1	85,24	-1,23	-2,37
YOR027W	STI1	85,20	-4,53	-7,61
YCL005W		85,08	1,04	-0,21
YGL066W	SGF73	85,06	4,29	-0,59
YPR063C		85,05	-0,51	-4,56
YDR377W	ATP17	84,99	-1,85	-3,29
YLR247C		84,94	-1,65	-3,68
YPR101W	SNT309	84,93	-0,38	-6,14
YPL002C	SNF8	84,85	3,24	5,47
YOL061W	PRS5	84,81	0,10	-2,49
YJL080C	SCP160	84,72	13,47	11,46
YOR069W	VPS5	84,67	-0,89	-2,79

Supplementary Table S2

YPR046W	MCM16	84,63	-7,42	-9,45
YGL104C	VPS73	84,60	0,10	-1,76
YPL023C	MET12	84,59	2,80	-1,06
YDL149W	ATG9	84,55	4,39	1,62
YMR078C	CTF18	84,55	0,99	-0,70
YMR035W	IMP2	84,54	-2,43	-5,56
YLR087C	CSF1	84,52	-2,97	-5,10
YIL124W	AYR1	84,43	-0,49	1,08
YOL030W	GAS5	84,34	-2,77	-4,75
YLR403W	SFP1	84,19	40,02	-6,65
YJR140C	HIR3	84,15	-3,74	-8,38
YPR044C		84,07	-7,55	-13,60
YDR226W	ADK1	83,97	6,09	3,16
YMR179W	SPT21	83,85	-3,53	-6,55
YER061C	CEM1	83,83	3,58	2,08
YIL018W	RPL2B	83,73	1,46	1,73
YGL214W		83,58	-1,59	-4,16
YPR076W		83,56	2,54	0,10
YHR064C	SSZ1	83,51	1,28	-3,94
YMR185W		83,45	1,25	-0,42
YLR013W	GAT3	83,44	-2,67	-5,04
YDR363W	ESC2	83,44	3,92	2,65
YJR105W	ADO1	83,37	-2,67	-4,33
YDR255C	RMD5	83,37	9,00	6,35
YDR469W	SDC1	83,33	-4,76	-6,11
YDL173W		83,32	3,65	1,42
YLR338W		83,23	-9,02	-6,37
YPL226W	NEW1	83,13	0,20	-0,30
YCR071C	IMG2	82,97	2,68	-2,99
YGR036C	CAX4	82,94	0,25	-1,11
YEL052W	AFG1	82,89	0,60	-0,40
YHR010W	RPL27A	82,89	5,16	4,06
YLR011W	LOT6	82,83	-1,02	-3,68
YBR026C	ETR1	82,82	1,69	7,38
YPR012W		82,80	-6,00	-9,44
YJR128W		82,78	1,85	-0,97
YMR142C	RPL13B	82,76	6,69	-1,78
YDR523C	SPS1	82,62	-2,65	-24,88
YLR271W		82,49	-3,59	-5,19
YMR145C	NDE1	82,48	-0,68	-3,30
YOR042W	CUE5	82,43	-5,18	-8,56
YDL041W		82,43	-4,88	-7,32
YGR197C	SNG1	82,34	3,96	2,41
YLR085C	ARP6	82,28	-0,10	-2,98
YER122C	GLO3	82,24	0,10	-2,80
YOR312C	RPL20B	82,21	4,77	1,75
YLR402W		82,19	2,57	-3,53
YMR048W	CSM3	82,16	3,96	-0,19
YJR033C	RAV1	82,15	-5,34	-6,47
YLL045C	RPL8B	82,09	0,00	-4,38
YNL215W	IES2	82,08	-1,08	-4,03
YLR255C		82,02	1,68	-1,58
YPL035C		81,83	-0,20	-2,23

Supplementary Table S2

YPL025C		81,82	3,98	0,80
YGR285C	ZUO1	81,72	9,16	2,23
YKL081W	TEF4	81,67	-1,98	-0,89
YOR076C	SKI7	81,66	2,11	-1,51
YOR297C	TIM18	81,65	-1,50	-4,09
YIL134W	FLX1	81,55	3,03	2,25
YER052C	HOM3	81,51	1,71	1,18
YIL146C	ECM37	81,50	1,26	3,97
YDR448W	ADA2	81,36	7,45	1,78
YGR004W		81,32	0,30	-2,17
YAL035W		81,29	24,27	15,56
YDR049W		81,28	-8,36	-10,50
YLR111W		81,23	1,00	0,20
YLR239C	LIP2	81,07	1,02	-3,65
YDR461W	MFA1	81,01	31,82	-11,56
YNL246W	VPS75	80,90	-7,01	-11,59
YJR074W	MOG1	80,89	3,98	-2,85
YLR337C	VRP1	80,89	-2,96	-0,30
YHR039C-B	VMA10	80,88	-1,40	-1,90
YIL157C		80,87	0,67	6,03
YOL029C		80,86	-2,63	-4,95
YPR040W	TIP41	80,84	2,98	-2,18
YNL323W	LEM3	80,77	-10,94	-8,79
YHL011C	PRS3	80,76	1,29	0,83
YKR073C		80,74	-0,76	-4,85
YDR140W		80,65	3,15	0,76
YLL032C		80,62	1,07	-2,93
YBL027W	RPL19B	80,58	4,87	1,89
YJR035W	RAD26	80,49	-0,88	-3,52
YAL013W		80,44	4,69	0,77
YLR262C	YPT6	80,43	-5,40	-6,70
YPL269W	KAR9	80,20	-2,40	-1,00
YDR257C	RMS1	80,20	8,67	7,05
YLR079W	SIC1	80,10	-1,66	-2,39
YOR073W		80,10	-4,05	-7,60
YHR178W	STB5	80,00	1,59	1,22
YGR272C		80,00	1,42	-1,14

Group C:

ORF	Name	%INH 4NQO 48h	%INH 4NQO 72h	%INH 4NQO 120h
YGL240W	DOC1	34,04	90,27	93,58
YJL096W	MRPL49	39,44	92,89	92,47
YDL202W	MRPL11	77,17	92,25	92,17
YGL129C	RSM23	22,88	91,49	91,99
YNL160W	YGP1	14,29	92,08	91,98
YML088W		53,37	91,99	91,89
YNL184C		22,77	91,72	91,72
YLR260W	LCB5	55,59	87,88	91,71
YGL107C	RMD9	10,68	90,52	91,67
YGL220W		33,58	90,92	91,63

Supplementary Table S2

YGR215W	RSM27	66,12	91,89	91,59
YLL009C	COX17	6,98	91,75	91,55
YMR184W		78,27	91,43	91,54
YMR267W	PPA2	23,08	91,53	91,53
YLR204W	QRI5	45,21	91,53	91,53
YHR038W	RRF1	73,35	91,72	91,52
YMR089C	YTA12	72,31	91,00	91,51
YGR076C	MRPL25	70,98	91,17	91,49
YMR282C	AEP2	52,69	91,38	91,48
YMR287C	MSU1	38,10	91,38	91,48
YML061C	PIF1	42,03	91,58	91,47
YMR286W	MRPL33	4,76	91,42	91,42
YHL038C	CBP2	50,27	90,93	91,42
YLR067C	PET309	43,88	91,31	91,42
YPR166C	MRP2	13,40	91,41	91,41
YJL209W	CBP1	25,22	91,39	91,39
YPL059W	GRX5	17,02	91,34	91,34
YGR171C	MSM1	9,78	91,34	91,34
YOL033W	MSE1	79,86	91,30	91,30
YGR150C		75,86	91,37	91,27
YBL044W		22,79	91,51	91,25
YPL029W	SUV3	46,67	91,21	91,21
YMR293C		20,20	91,01	91,13
YLR369W	SSQ1	15,31	91,17	91,06
YLR069C	MEF1	38,21	91,28	91,06
YHR147C	MRPL6	35,51	91,06	91,06
YMR024W	MRPL3	70,37	90,92	90,92
YLR139C	SLS1	45,86	91,00	90,89
YGL135W	RPL1B	32,82	89,51	90,83
YDR529C	QCR7	57,28	91,00	90,70
YMR071C	TVP18	58,41	89,64	90,68
YOR305W		62,84	90,78	90,67
YKR006C	MRPL13	47,06	90,78	90,67
YDL049C	KNH1	75,59	90,56	90,67
YKL155C	RSM22	37,93	90,49	90,49
YJL063C	MRPL8	8,25	90,67	90,46
YCR004C	YCP4	71,30	90,32	90,42
YPR099C		37,23	90,49	90,38
YBR163W	DEM1	15,57	90,24	90,33
YLL018C-A	COX19	18,10	90,53	90,33
YOL096C	COQ3	73,86	89,63	90,30
YBL100C		68,91	90,20	90,30
YDR078C	SHU2	68,63	89,94	90,23
YNL213C		58,37	90,44	90,23
YDR079W	PET100	42,37	90,08	90,18
YGR255C	COQ6	46,24	90,37	90,17
YLR312W-A	MRPL15	57,37	90,28	90,17
YKL138C	MRPL31	73,71	90,52	90,10
YDR298C	ATP5	71,08	89,88	90,10
YDR507C	GIN4	41,03	90,46	90,01
YGR180C	RNR4	27,78	90,13	89,87
YDR175C	RSM24	77,62	89,73	89,83
YGR220C	MRPL9	21,43	89,20	89,75

Supplementary Table S2

YDL063C		50,50	89,59	89,70
YHR011W	DIA4	58,45	90,67	89,59
YGR006W	PRP18	60,53	90,30	89,53
YMR066W	SOV1	48,04	88,83	89,47
YGR257C	MTM1	74,70	89,73	89,43
YKL169C		66,30	89,28	89,40
YPL148C	PPT2	6,74	89,45	89,33
YGR222W	PET54	11,61	89,06	89,29
YEL029C	BUD16	55,00	89,13	89,24
YAL048C		78,90	89,34	89,22
YNL252C	MRPL17	74,32	91,19	89,22
YER070W	RNR1	70,66	88,69	89,08
YDR518W	EUG1	38,67	90,98	88,96
YJL003W	COX16	8,65	89,55	88,90
YBR122C	MRPL36	77,76	88,91	88,81
YGR219W		15,13	88,23	88,81
YDR295C		72,86	88,77	88,77
YJR113C	RSM7	75,00	88,99	88,72
YDR042C		41,04	88,93	88,71
YDR197W	CBS2	76,08	88,51	88,51
YPR072W	NOT5	54,70	89,90	88,42
YDL062W		23,02	88,16	88,40
YDR065W		75,06	88,34	88,34
YDR114C		52,89	88,12	88,34
YKL016C	ATP7	57,62	88,29	88,29
YDL146W		11,40	88,08	88,20
YBL002W	HTB2	7,48	88,14	88,14
YJR004C	SAG1	73,14	90,26	87,73
YDL198C	YHM1	39,09	87,35	86,79
YDL181W	INH1	24,03	85,92	86,07
YJR144W	MGM101	25,83	85,69	85,85
YLR114C		1,19	85,93	85,76
YDR115W		73,43	83,20	83,52
YBL012C		28,57	83,36	83,36
YOR211C	MGM1	3,41	83,53	83,14
YBL090W	MRP21	12,80	83,18	81,64
YPR067W	ISA2	13,68	80,43	79,95
