# Supplementary material for: Genome-Wide Analysis of Factors Affecting Transcription Elongation and DNA Repair: A New Role for PAF and Ccr4-Not in Transcription-Coupled Repair
Source: PLoS Genet. 2009 Feb 6;5(2):e1000364. doi: 10.1371/journal.pgen.1000364 (PMC2629578; doi:10.1371/journal.pgen.1000364)
Supplement: Table S3 — Genetic analysis of sensitivity to DNA-damaging agents. Thirty 4-NQO-sensitive strains of group A that encompassed mutations of the SAGA, PAF, CDKs, and Mediator complexes as well as proteins involved in RNAPII transcription, mRNA processing and degradation, chromatin remodeling, DNA-damage response, and translation initiation were analyzed for drug sensitivity. The indicated strains were spotted as 10-fold serial dilutions on complete minimal medium (SC) and minimal medium containing 0.1 µg/ml 4-nitroquinoline-N-oxide (4-NQO), 0,015% methyl methane sulfonate (MMS), or 0.1 nM menadione (Mnd). UV sensitivity (UV) was assessed following irradiation with 70 J/m2. Plates were grown for 3 days at 30°C or 37°C and cell viability for each condition was scored as + for growth as wild-type, as +/− for moderate growth defects, and as - for severe growth defects. Most of the UV sensitive strains were also sensitive to 4-NQO, consistent with the fact that the bulky adduct produced by 4-NQO get repaired by NER (exceptions are ctk2, spt5, bdf1, lsm1, npl6, and fps1). However, 9 of the 4-NQO sensitive strains were not sensitive to UV (spt20, bur2, med2, med16, med20, hpr1, spt4, rpb9, and swi6), indicating that 4-NQO is more than a ‘UV-mimetic’ agent. Those strains appeared mainly sensitive to Mnd, reflecting their deficiencies in the presence of oxidative damage. Comparison of strain sensitivity to 4-NQO, MMS, Mnd, and heat stress did not lead to significant clustering of cross-resistance, indicating that each drug leads to its own response, as observed previously for 15 DNA-damaging agents including MMS and 4-NQO [21],[82]. (0.09 MB DOC) [file pgen.1000364.s004.doc]

**Supplementary Table S3**

***Genetic analysis of sensitivity to DNA-damaging agents***

|  | **strains** | **SC** | **4NQO** | **UV** | **MMS** | **Mnd** | **37°C** |
| --- | --- | --- | --- | --- | --- | --- | --- |
|  | *WT* | + | + | + | + | + | + |
| **SAGA** | *spt8* | + | + | + | + | +/- | + |
|  | *spt20* | +/- | - | +/- | - | +/- | - |
| **PAF** | *cdc73* | + | +/- | +/- | - | +/- | +/- |
| **CDKs** | *ctk1* | + | + | + | + | +/- | + |
|  | *ctk2* | + | + | +/- | + | - | + |
|  | *ctk3* | + | +/- | +/- | + | +/- | + |
|  | *bur2* | +/- | - | +/- | - | - | - |
| **Mediator** | *med2* | +/- | - | +/- | - | - | - |
|  | *med3* | +/- | +/- | +/- | - | - | - |
|  | *med16* | + | +/- | + | +/- | - | - |
|  | *med20* | + | - | + | + | - | +/- |
| **RNAPII transcription** | *rpb9* | +/- | - | +/- | - | +/- | - |
|  | *spt4* | + | +/- | + | +/- | + | + |
|  | *spt5* | + | + | +/- | +/- | + | + |
|  | *swi6* | + | +/- | + | - | +/- | + |
|  | *bdf1* | + | + | +/- | + | + | + |
|  | *elc1* | + | + | + | + | - | + |
|  | *taf14* | + | + | + | - | +/- | - |
| **mRNA biogenesis and export** | *tho2* | + | - | +/- | +/- | - | + |
|  | *hpr1* | + | +/- | + | +/- | - | + |
|  | *thp1* | + | +/- | +/- | + | - | + |
| **mRNA degradation** | *lsm1* | + | + | +/- | +/- | +/- | + |
|  | *lsm7* | + | +/- | +/- | + | - | + |
| **Chromatin remodeling** | *ies6* | + | - | +/- | - | +/- | - |
|  | *npl6* | + | + | +/- | + | + | - |
| **damage response** | *rad4* | + | - | - | - | + | +/- |
|  | *def1* | + | - | +/- | - | +/- | +/- |
|  | *yap1* | + | + | + | + | +/- | + |
| **others** | *fps1* | + | + | +/- | +/- | +/- | - |
|  | *gcn2* | +/- | +/- | +/- | - | +/- | - |
