# Supplementary material for: Genome-Wide Analysis of Factors Affecting Transcription Elongation and DNA Repair: A New Role for PAF and Ccr4-Not in Transcription-Coupled Repair
Source: PLoS Genet. 2009 Feb 6;5(2):e1000364. doi: 10.1371/journal.pgen.1000364 (PMC2629578; doi:10.1371/journal.pgen.1000364)
Supplement: Table S4 — Analysis of mutant strains leading to MPA sensitivity. Growth was monitored after 72 hours in MPA-containing media (25 µg/ml). Strains showing at least 50% growth inhibition are listed together with their GLAM-ratios. Values above 60% inhibition and ratios below 0.5 are highlighted in red. n.d., not determined. (0.03 MB PDF) [file pgen.1000364.s005.pdf]

# Supplementary Table S4

| ORF | Name | %INH MIC | Screen Daignan-Fornier B. | GLAM ratio PHO5-lacZ/PHO5 | GLAM ratio PHO5-LAC4/PHO5 |
|---|---|---|---|---|---|
| YIR016W | | 95,83 | No | 0.52 | 0.73 |
| YCR053W | THR4 | 89,09 | No | n.d. | n.d. |
| YHL031C | GOS1 | 88,95 | No | n.d. | n.d. |
| YGR018C | | 88,80 | No | 1.16 | 0.88 |
| YBR081C | SPT7 | 88,57 | Yes | 0.59 | 0.67 |
| YBR101C | FES1 | 88,54 | No | 0.51 | 0.72 |
| YML008C | ERG6 | 88,12 | Yes | n.d. | n.d. |
| YOR184W | SER1 | 88,11 | Yes | n.d. | n.d. |
| YER051W | JHD1 | 88,00 | No | 0.85 | 1.13 |
| YER083C | RMD7 | 87,85 | No | 0.85 | 0.96 |
| YOL148C | SPT20 | 87,79 | Yes | **0.16** | **0.29** |
| YDR483W | KRE2 | 87,65 | No | n.d. | n.d. |
| YJL140W | RPB4 | 86,99 | Yes | **0.03** | **0.08** |
| YHR025W | THR1 | 86,74 | Yes | n.d. | n.d. |
| YGL244W | RTF1 | 86,69 | Yes | **0.30** | **0.34** |
| YBL058W | SHP1 | 86,34 | Yes | 0.57 | **0.46** |
| YGL070C | RPB9 | 86,12 | Yes | **0.46** | **0.49** |
| YMR272C | SCS7 | 86,05 | No | 0.86 | 0.94 |
| YLR055C | SPT8 | 85,83 | Yes | 0.86 | 0.63 |
| YGL042C | | 85,77 | Yes | 0.66 | 0.82 |
| YER052C | HOM3 | 85,48 | No | n.d. | n.d. |
| YGR208W | SER2 | 85,29 | Yes | n.d. | n.d. |
| YNL229C | URE2 | 84,21 | No | 0.94 | 0.51 |
| YML112W | CTK3 | 84,13 | Yes | 1.38 | 0.88 |
| YDR392W | SPT3 | 83,55 | Yes | **0.41** | **0.47** |
| YDL074C | BRE1 | 83,10 | Yes | **0.37** | **0.47** |
| YKL212W | SAC1 | 82,99 | Yes | n.d. | n.d. |
| YLR226W | BUR2 | 81,97 | No | **0.08** | **0.30** |
| YDR158W | HOM2 | 81,86 | Yes | n.d. | n.d. |
| YGL012W | ERG4 | 81,69 | Yes | n.d. | n.d. |
| YBR097W | VPS15 | 81,62 | Yes | n.d. | n.d. |
| YOL051W | MED15 | 81,51 | No | **0.38** | **0.40** |
| YJR139C | HOM6 | 81,46 | Yes | n.d. | n.d. |
| YMR202W | ERG2 | 81,32 | No | n.d. | n.d. |
| YGL043W | DST1 | 81,28 | Yes | 0.62 | 0.85 |
| YIR014W | | 81,26 | No | 0.81 | 1.02 |
| YBR200W | BEM1 | 81,13 | Yes | n.d. | n.d. |
| YAL035W | FUN12 | 80,88 | Yes | **0.43** | 0.75 |
| YDR207C | UME6 | 80,11 | No | 0.84 | 0.99 |
| YPR072W | NOT5 | 78,23 | No | **0.25** | **0.36** |
| YGL038C | OCH1 | 78,06 | Yes | n.d. | n.d. |
| YCR081W | MED12 | 77,00 | No | 0.59 | 0.60 |
| YLR337C | VRP1 | 76,37 | Yes | 0.67 | 0.64 |
| YPL230W | | 76,15 | No | **0.43** | **0.48** |
| YPL055C | LGE1 | 74,43 | No | 0.54 | **0.41** |
| YMR304W | UBP15 | 73,65 | No | **0.44** | 1.08 |
| YER014C-A | BUD25 | 73,23 | No | n.d. | n.d. |
| YNL139C | THO2 | 73,14 | No | **0.04** | **0.13** |
| YOL086C | ADH1 | 72,96 | No | n.d. | n.d. |
| YLR148W | PEP3 | 72,03 | Yes | n.d. | n.d. |

# Supplementary Table S4

| | | | | | |
|---|---|---|---|---|---|
| YGR063C | SPT4 | 71,33 | No | **0.14** | **0.13** |
| YDL005C | MED2 | 69,67 | No | **0.31** | **0.35** |
| YPL045W | VPS16 | 68,87 | No | n.d. | n.d. |
| YBR069C | TAT1 | 68,72 | Yes | n.d. | n.d. |
| YER014W | HEM14 | 68,64 | No | n.d. | n.d. |
| YLR418C | CDC73 | 68,08 | No | **0.23** | 0.56 |
| YAL016W | TPD3 | 67,99 | No | **0.35** | 0.58 |
| YGL058W | RAD6 | 67,00 | Yes | 0.56 | **0.44** |
| YGR204W | ADE3 | 66,19 | No | n.d. | n.d. |
| YHL025W | SNF6 | 66,16 | Yes | 0.82 | 0.59 |
| YLR338W | OPI9 | 65,60 | Yes | n.d. | n.d. |
| YER086W | ILV1 | 65,07 | Yes | n.d. | n.d. |
| YJL175W | | 63,58 | Yes | 0.74 | 1.24 |
| YLR056W | ERG3 | 61,95 | Yes | n.d. | n.d. |
| YDR320C | SWA2 | 61,93 | No | n.d. | n.d. |
| YDL100C | ARR4 | 61,56 | Yes | n.d. | n.d. |
| YMR231W | PEP5 | 61,34 | Yes | n.d. | n.d. |
| YOR036W | PEP12 | 59,45 | Yes | n.d. | n.d. |
| YCL007C | CWH36 | 58,01 | No | n.d. | n.d. |
| YAL021C | CCR4 | 57,71 | No | **0.46** | 0.64 |
| YLR318W | EST2 | 57,14 | No | **0.22** | **0.04** |
| YGL223C | COG1 | 56,96 | No | n.d. | n.d. |
| YGR038W | ORM1 | 55,91 | No | 0.65 | 0.94 |
| YLR239C | LIP2 | 54,93 | No | 0.77 | **0.31** |
| YKL139W | CTK1 | 53,85 | Yes | 1.39 | 1.53 |
| YML095C | RAD10 | 53,71 | No | 0.85 | 1.08 |
| YJR018W | | 53,63 | Yes | **0.10** | **0.34** |
| YKR001C | VPS1 | 52,84 | No | n.d. | n.d. |
| YBR021W | FUR4 | 51,79 | No | n.d. | n.d. |
| YPR160W | GPH1 | 51,06 | No | n.d. | n.d. |
| YDR323C | PEP7 | 51,00 | Yes | n.d. | n.d. |
| YAL002W | VPS8 | 50,83 | No | n.d. | n.d. |
| YPL084W | BRO1 | 50,58 | No | 0.51 | 0.74 |
| YCL008C | STP22 | 50,20 | No | n.d. | n.d. |
